# Diagnostic Discrimination of the Cardiometabolic Index for Prevalent Metabolic Syndrome in Postmenopausal Women With Severe Obesity: A Cross‐Sectional Study

**DOI:** 10.1002/hsr2.73503

**Published:** 2026-11-10

**Authors:** Daniel de Luis, Olatz Izaola, Juan José Lopez Gomez, Daniel Rico, David Primo

**Affiliations:** ^1^ Servicio de Endocrinología y Nutrición, Hospital Clínico Universitario de Valladolid Valladolid Spain; ^2^ Valladolid Health Research Institute (IBioVALL) Valladolid Spain; ^3^ Centro de Investigación de Endocrinología y Nutrición Fac Medicina Universidad de Valladolid Valladolid Spain; ^4^ Centro de Investigación Biomedica en Red (CIBEROBN) de la Obesidad y Nutrición. Instituto de Salud Carlos III Madrid Spain

**Keywords:** cardiometabolic index, homeostatic model assessment, metabolic syndrome, obesity, postmenopausal

## Abstract

**Background:**

The cardiometabolic index (CMI), combining waist‐to‐height ratio and the triglyceride‐to‐HDL cholesterol ratio, shares components with the diagnostic definition of metabolic syndrome (MetS). Its apparent discrimination therefore requires cautious interpretation.

**Aim:**

To assess the association of CMI with prevalent MetS and describe its diagnostic discrimination alongside HOMA‐IR in postmenopausal women with severe obesity.

**Methods:**

In this cross‐sectional study, anthropometric measures, blood pressure, fasting biochemical variables and MetS status were assessed concurrently in 499 postmenopausal women referred for evaluation of severe obesity (BMI > 35 kg/m^2^). CMI = [triglycerides (mmol/L)/HDL cholesterol (mmol/L)] × [waist circumference/height], with both lengths expressed in the same unit. ROC analysis and the Youden index were used to describe discrimination and select cut‐offs.

**Results:**

MetS was present in 300 women (60.1%). The reported association above versus below the median CMI of 0.74 was OR 2.65 (95% CI 1.99–3.53); its adjustment status and source analysis require verification. The reported AUCs were 0.76 (95% CI 0.71–0.80) for CMI and 0.70 (95% CI 0.66–0.81) for HOMA‐IR. At the reported cut‐offs of 0.84 and 2.64, respectively, sensitivities were 85.6% and 64.9%, and specificities 72.5% and 69.7%.

**Conclusions:**

CMI was associated with prevalent MetS in this selected cross‐sectional sample. Component overlap with the MetS definition may affect apparent discrimination.

## 1 Introduction

Metabolic syndrome (MetS) is a cluster of cardiovascular risk factors associated with adverse outcomes [1, 2, 3]. In a cross‐sectional study, concurrent measurement of an index and MetS can assess association and discrimination of prevalent disease, but cannot establish prospective prediction.

Body mass index (BMI) and waist circumference are commonly used anthropometric measures; BMI does not distinguish fat from lean mass or directly characterize fat distribution. Composite measures may describe concurrent metabolic phenotypes, although their interpretation depends on their relationship to the outcome definition. The prevalence of MetS tends to rise after menopause [4]. Age, changes in body composition, lifestyle, and hormonal changes may all contribute; observational comparisons do not establish menopause as an independent causal factor [5, 6, 7, 8, 9, 10, 11, 12, 13, 14, 15].

The cardiometabolic index (CMI) combines waist‐to‐height ratio with the triglyceride‐to‐HDL cholesterol ratio. Studies in women with severe obesity have examined its discrimination for prevalent MetS [16], but evidence specific to postmenopausal women referred for evaluation of severe obesity remains limited. HOMA‐IR was chosen as a comparator because insulin resistance is closely related to MetS and HOMA‐IR uses fasting glucose and insulin rather than the lipid and waist components used to define MetS [16, 17, 18, 19].

In this context, CMI includes triglycerides, HDL cholesterol and a waist measure, which also contribute to the ATP III definition of MetS. This incorporation overlap can increase apparent association and discrimination even without independent clinical information. We therefore interpret the analysis as identification of prevalent MetS in this selected population, not validation of a new risk predictor.

We aimed to describe the association between CMI and prevalent MetS and its apparent diagnostic discrimination in postmenopausal women with severe obesity, with HOMA‐IR as a prespecified descriptive comparator.

## 2 Patients and Methods

### 2.1 Patients

A cross‐sectional observational study was conducted within a Health Area in Spain. A total of 499 participants were recruited using a non‐probabilistic consecutive sampling approach. The study population consisted exclusively of Caucasian postmenopausal women with obesity who had been referred from Primary Care Health Departments—covering both rural and urban settings—to a tertiary hospital for specialized evaluation. These patients were attended at the hospital outpatient clinic for a comprehensive assessment of severe obesity, defined as a BMI greater than 35 kg/m^2^. This was a specialist referral sample and is not representative of the general population.

All participants provided written informed consent prior to inclusion in the study. The investigation was performed in accordance with the principles of the Declaration of Helsinki, and the study protocol received approval from the institutional Ethics Committee (registration code 06/2020). Eligibility criteria included the presence of severe obesity (BMI > 35 kg/m^2^) and a confirmed postmenopausal status, defined as the absence of menstruation for at least 12 consecutive months without any underlying pathological cause, without hormonal confirmation. Exclusion criteria comprised the presence of severe comorbid conditions, such as chronic kidney disease, chronic liver disease, heart failure, previous cardiovascular events, or active malignancy. In addition, women with excessive alcohol consumption (> 10 ethanol units per day; 1 unit = 14 g ethanol) were excluded. Participants were also excluded if they had followed a hypocaloric diet within the preceding 6 months or were receiving pharmacological treatments that could influence metabolic parameters, including statin and fibrate users, antihypertensive treatment and treatment for diabetes.

Data collection was carried out between December 2020 and December 2024 by a team of trained investigators. Collected variables included sociodemographic characteristics, anthropometric and body composition parameters (body weight, BMI, fat mass assessed by bioelectrical impedance, and waist circumference), blood pressure measurements, and biochemical markers. During the same clinical visit, fasting venous blood samples (5 mL) were obtained after a minimum 10‐h overnight fast. Samples were collected in ethylenediaminetetraacetic acid (EDTA)‐containing tubes and processed for subsequent biochemical analyses.

#### 2.1.1 Metabolic Syndrome, Adiposity Parameters, and Blood Pressure

MetS was defined using the stated ATP III criteria [20] as at least three of the following: waist circumference > 88 cm; triglycerides > 150 mg/dL; HDL cholesterol < 50 mg/dL; blood pressure ≥ 130/85 mmHg; and fasting glucose > 110 mg/dL. Statin and fibrate users, antihypertensive treatment and treatment for diabetes were excluded.

To ensure consistency and reduce interobserver variability, all anthropometric and adiposity‐related measurements were performed by the same trained investigator. Waist circumference was assessed using a standardized flexible tape (Omron, LA, CA, USA), positioned midway between the lower rib margin and the iliac crest. Two measurements were obtained for each participant, and the mean value was considered for analysis. Height was measured in centimetres using a calibrated stadiometer (Omron, LA, CA, USA), while body weight was recorded using digital scales under standardized conditions, with participants wearing light clothing and no footwear. BMI was calculated as weight (kg) divided by height squared (m^2^). The CMI was calculated using the following formula [3]: CMI = [triglycerides (mmol/L)/HDL cholesterol (mmol/L)] × waist‐to‐height ratio (WHtR). Total body fat mass was estimated by bioelectrical impedance analysis (BIA) on fasting condition using a high‐precision device (EFG BIA 101 Anniversary, Akern, Italy), with an accuracy of 5 g [21]. Blood pressure was measured using a validated sphygmomanometer (Omron, LA, CA, USA) after a 10‐min seated rest period. A total of four measurements were recorded for each participant; the first reading was excluded, and the mean of the remaining three measurements was used for statistical analysis. The lipid ratio uses triglycerides and HDL cholesterol both in mmol/L. Values were initially recorded in mg/dL; each analyte must first be converted separately (triglycerides ÷88.57; HDL cholesterol ÷38.67). Waist circumference and height must be in identical length units. Because these variables overlap with MetS components, CMI discrimination for this outcome is susceptible to incorporation bias.

#### 2.1.2 Biochemical Procedures

Biochemical analyses were performed on fasting samples using the automated analyzer described above. Glucose, insulin, C‐reactive protein, total cholesterol, HDL cholesterol and triglycerides were measured with the COBAS INTEGRA 400 (Roche Diagnostics, Basel, Switzerland). LDL cholesterol was calculated using the Friedewald formula when applicable [22]. HOMA‐IR is fasting glucose (mg/dL) × fasting insulin (µIU/mL)/405 [23].

Circulating adipokines were quantified using enzyme‐linked immunosorbent assays (ELISA). Serum adiponectin levels were measured using commercially available kits from R&D Systems Inc. (Minnesota, USA), with a reference range of 8.65–21.43 µg/mL. Resistin concentrations were determined using kits from Biovendor Laboratory Inc. (Brno, Czech Republic), with a reference range of 4–12 ng/mL, and leptin levels were measured using kits from Diagnostic Systems Laboratories Inc. (Texas, USA), with a reference range of 10–100 ng/mL.

### 2.2 Statistical Evaluation

For estimation of the ROC area, a sample of approximately 400 participants (240 with and 160 without MetS, assuming a prevalence of 60%) would provide an approximate 95% confidence interval with a half‐width of 0.05 around an anticipated AUC of 0.70, using the Hanley–McNeil variance method. Normally distributed variables were reported as mean ± SD, whereas non‐normally distributed variables were presented as median and interquartile range. Categorical variables were compared using Pearson's chi‐square test; Student's t‐test or the Mann–Whitney U test was used as appropriate for continuous variables. Spearman correlations were used for associations. ROC AUC describes discrimination across thresholds. The reported cut‐offs were selected with the Youden index (sensitivity + specificity − 1). A paired comparison of CMI and HOMA‐IR AUCs (e.g., DeLong's test), component‐wise analyses and 95% confidence intervals for sensitivity, specificity and likelihood ratios was performed. Tests were two‐sided with a nominal *p* < 0.05.

## 3 Results

The baseline clinical, anthropometric, and biochemical characteristics of the study population according to MetS status are shown in Table 1. A total of 499 postmenopausal women with severe obesity were included in the analysis, with a mean age of 60.1 ± 7.5 years (range: 50–81 years). Among them, 300 individuals (60.1%) fulfilled the diagnostic criteria for MetS, whereas 199 (39.9%) did not. Significant differences in anthropometric parameters were observed between groups.

**Table 1:** Clinical and biochemical characteristics according to metabolic syndrome status.

Parameters	Total (*n* = 499)	Without MetS (*n* = 199)	MetS (*n* = 300)	*p*
BMI (kg/m^2^)	38.7 (1.2)	37.5 (0.9)	39.7 (0.7)	0.02
Body Weight (kg)	90.8 (9.3)	84.3 (9.1)	95.1 (6.0)	0.02
Total Fat Mass (kg)	41.7 (11.4)	36.7 (3.2)	45.3 (4.2)	0.03
WC (cm)	113.6 (3.1)	107.1 (4.2)	118.7 (3.2)	0.01
SBP (mmHg)	136.0 (7.3)	128.7 (4.3)	141.2 (4.0)	0.01
DBP (mmHg)	85.2 (3.3)	82.0 (4.1)	87.4 (3.3)	0.03
Fasting glucose (mg/dL)	108.8 (6.4)	99.3 (8.2)	116.3 (5.1)	0.01
Total cholesterol (mg/dL)	222.8 (12.1)	223.3 (9.2)	224.2 (8.4)	0.27
LDL‐cholesterol (mg/dL)	139.0 (23.5)	135.1 (6.3)	142.2 (6.2)	0.25
HDL‐cholesterol (mg/dL)	59.8 (2.2)	60.8 (2.2)	57.4 (1.1)	0.03
Triglycerides (mg/dL)	124.7 (9.4)	107.6 (8.3)	137.2 (6.9)	0.02
Insulin (µUI/L)	12.6 (1.3)	10.5 (2.2)	14.9 (0.4)	0.02
HOMA‐IR	3.4 (0.9)	2.4 (0.5)	4.2 (0.2)	0.02
CRP (mg/dL)	5.5 (5.1–5.9)†	5.3 (4.7–5.9)†	6.0 (5.3–6.7)†	0.28
Resistin (ng/ml)	5.0 (4.9–5.1)†	4.6 (4.3–4.9)†	5.1 (4.5–5.7)†	0.39
Adiponectin (ng/ml)	25.3 (22.9–27.7)†	33.7 (31.1–36.3)†	20.3 (19.6–21.0)†	0.02
Leptin (ng/ml)	72.2 (67.3–77.1)†	58.1 (56.5–59.7)†	80.0 (77.3–82.7)†	0.02
CMI	0.88 ± 0.3	0.81 ± 0.3	0.94 ± 0.1	0.02

Abbreviations: BMI, body mass index; CMI, cardiometabolic index; CRP, C‐reactive protein; DBP, diastolic blood pressure; HDL, high‐density lipoprotein; HOMA‐IR, homeostasis model assessment of insulin resistance; LDL, low‐density lipoprotein; MetS, metabolic syndrome; SBP, systolic blood pressure; WC, waist circumference. Number (SD). Number (Q1–Q3)†

Participants with MetS exhibited higher values of BMI compared to those without MetS (Δ = 2.2 kg/m^2^; *p* = 0.02). Similarly, body weight was significantly increased in the MetS group (Δ = 10.8 kg; *p* = 0.02), as well as total fat mass (Δ = 8.6 kg; *p* = 0.03). Waist circumference also showed a marked increase in individuals with MetS (Δ = 11.6 cm; *p* = 0.01), highlighting the strong association between central adiposity and metabolic risk. Blood pressure values were significantly higher in participants with MetS. Systolic blood pressure was increased (Δ = 12.5 mmHg; *p* = 0.01), as well as diastolic blood pressure (Δ = 5.4 mmHg; *p* = 0.03), confirming the clustering of cardiovascular risk factors in this group (Table 1).

Regarding biochemical parameters, individuals with MetS displayed a significantly altered metabolic profile. Fasting glucose levels were higher in the MetS group (Δ = 17.0 mg/dL; *p* = 0.01). Triglyceride concentrations were also significantly elevated (Δ = 29.6 mg/dL; *p* = 0.02), while HDL‐cholesterol levels were significantly lower (Δ = −3.4 mg/dL; *p* = 0.03). Markers of insulin resistance were significantly increased in the MetS group, with higher insulin levels (Δ = 4.4 µUI/L; *p* = 0.02) and HOMA‐IR values (Δ = 1.8 units; *p* = 0.02). In addition, leptin concentrations were significantly elevated (Δ = 21.9 ng/mL; *p* = 0.02), whereas adiponectin levels were significantly reduced (Δ = −13.4 ng/mL; *p* = 0.02). No statistically significant differences were observed between groups for total cholesterol, LDL‐cholesterol, CRP, or resistin levels. The CMI was significantly higher in participants with MetS compared to those without MetS (0.94 ± 0.1 vs. 0.81 ± 0.09; Δ = 0.13 units; *p* = 0.02), supporting its role as a marker of metabolic dysfunction.

The prevalence of individual MetS components is summarized in Table 2. As expected, all diagnostic criteria were significantly more frequent in the MetS group compared to the non‐MetS group. Central obesity was present in 95.8% of individuals with MetS versus 68.7% without MetS (*p* = 0.02). Hypertriglyceridemia was observed in 18.3% versus 4.6% (*p* = 0.01), low HDL‐cholesterol in 23.3% versus 7.5% (*p* = 0.02), hypertension in 82.5% versus 26.3% (*p* = 0.001), and hyperglycemia in 50.2% versus 3.6% (*p* = 0.001).

**Table 2:** Descriptive frequency of ATP III metabolic syndrome components by MetS status.

Parameters	Total group (*n* = 499)	No MetS (*n* = 199)	MetS (*n* = 300)	*p*
Metabolic syndrome	60.1%	0%	100%	0.001
Central obesity	85.0%	68.7%	95.8%	0.02
Hypertriglyceridemia	12.8%	4.6%	18.3%	0.01
Low HDL cholesterol	17.0%	7.5%	23.3%	0.02
Hypertension	60.1%	26.3%	82.5%	0.001
Hyperglycemia	31.7%	3.6%	50.2%	0.001

*Note:* Cut‐off values for the diagnostic criteria included central obesity (waist circumference > 88 cm), hypertension (systolic blood pressure ≥ 130 mmHg and/or diastolic blood pressure ≥ 85 mmHg or ongoing antihypertensive treatment), hypertriglyceridemia (triglycerides > 150 mg/dL or lipid‐lowering therapy), and hyperglycemia (fasting plasma glucose > 110 mg/dL or pharmacological treatment). Differences between groups were considered statistically significant at *p* < 0.05.

Table 3 presents exploratory correlations. The CMI–waist correlation (*r* = 0.96) is partly mathematical because waist‐to‐height ratio is included in CMI; it cannot establish an independent relationship with visceral adiposity. Similarly, CMI contains triglycerides and HDL cholesterol. The weak correlation with CRP (*r* = 0.22) does not establish an inflammatory mechanism.

**Table 3:** Correlation analysis of CMI and HOMA‐IR with biochemical and anthropometric parameters.

Parameters	Total group *n* = 499	No metabolic syndrome *n* = 199	Metabolic syndrome *n* = 300	Total group *n* = 499	No metabolic syndrome *n* = 199	Metabolic syndrome *n* = 300
	**CMI Index**			**HOMA‐IR**		
Waist circumference (cm)	*r* = 0.96, *p* = 0.001	*r* = 0.95, *p* = 0.001	*r* = 0.96, *p* = 0.001	*r* = 0.39, *p* = 0.01	*r* = 0.31, *p* = 0.02	*r* = 0.40, *p* = 0.001
Total fat mass (kg)	*r* = 0.55, *p* = 0.002	*r* = 0.21, *p* = 0.03	*r* = 0.68, *p* = 0.001	*r* = 0.35, *p* = 0.01	*r* = 0.27, *p* = 0.01	*r* = 0.36, *p* = 0.001
Body weight (kg)	*r* = 0.69, *p* = 0.001	*r* = 0.40, *p* = 0.02	*r* = 0.72, *p* = 0.001	*r* = 0.37, *p* = 0.01	*r* = 0.26, *p* = 0.01	*r* = 0.47, *p* = 0.001
HOMA‐IR	*r* = 0.38, *p* = 0.001	*r* = 0.30, *p* = 0.001	*r* = 0.49, *p* = 0.001	—	—	—
Insulin (µUI/L)	*r* = 0.36, *p* = 0.002	*r* = 0.26, *p* = 0.002	*r* = 0.39, *p* = 0.001	*r* = 0.97, *p* = 0.001	*r* = 0.96, *p* = 0.001	*r* = 1.00, *p* = 0.001
Triglycerides (mg/dL)	*r* = 0.21, *p* = 0.01	*r* = 0.19, *p* = 0.01	*r* = 0.34, *p* = 0.001	*r* = 0.30, *p* = 0.01	*r* = 0.21, *p* = 0.02	*r* = 0.32, *p* = 0.01
HDL‐Cholesterol (mg/dL)	*r* = −0.17, *p* = 0.01	*r* = −0.09, *p* = 0.12	*r* = −0.27, *p* = 0.01	*r* = −0.17, *p* = 0.01	*r* = −0.13, *p* = 0.02	*r* = −0.17, *p* = 0.001
CRP (mg/dL)	*r* = 0.22, *p* = 0.01	*r* = 0.14, *p* = 0.01	*r* = 0.24, *p* = 0.001	*r* = 0.12, *p* = 0.01	*r* = 0.11, *p* = 0.02	*r* = 0.15, *p* = 0.03
Glucose (mg/dL)	*r* = 0.29, *p* = 0.01	*r* = 0.13, *p* = 0.12	*r* = 0.37, *p* = 0.01	*r* = 0.51, *p* = 0.001	*r* = 0.49, *p* = 0.001	*r* = 0.55, *p* = 0.001

*Note:* Statistical differences between groups (*p* < 0.05).

Abbreviations: CMI, Cardiometabolic index; CRP, C reactive protein; CRP, C reactive protein; HOMA‐IR, homeostasis model assessment.

Receiver operating characteristic (ROC) curve analysis for the identification of MetS is shown in Table 4. The area under the curve (AUC) for HOMA‐IR was 0.70 (95% CI: 0.66–0.81; *p* = 0.01), indicating moderate discriminative ability. The optimal cut‐off value determined by the Youden index was 2.64, yielding a sensitivity of 64.9% and specificity of 69.7%, with a positive likelihood ratio (LR + ) of 2.15 and a negative likelihood ratio (LR − ) of 0.50.

**Table 4:** Diagnostic performance of HOMA‐IR and CMI for metabolic syndrome and its individual components.

Outcome	Index	AUC (95% CI)	Cut‐off	Sensitivity (%)	Specificity (%)	p value*	LR +	LR −
Metabolic syndrome	HOMA‐IR	0.70 (0.66–0.81)	2.64	64.9	69.7	0.018	2.14	0.50
	CMI	0.76 (0.71–0.80)	0.84	85.6	72.5		1.80	0.28
Central obesity	HOMA‐IR	0.63 (0.58–0.68)	2.58	61.8	60.9	< 0.001	1.58	0.63
	CMI	0.79 (0.75–0.83)	0.72	78.4	70.6		2.67	0.31
Hypertriglyceridemia	HOMA‐IR	0.66 (0.61–0.71)	2.71	65.2	62.8	< 0.001	1.75	0.55
	CMI	0.86 (0.82–0.89)	0.82	82.1	78.9		3.89	0.23
Low HDL cholesterol	HOMA‐IR	0.64 (0.59–0.69)	2.69	63.5	61.7	< 0.001	1.66	0.59
	CMI	0.84 (0.80–0.88)	0.79	80.6	76.8		3.47	0.25
Hypertension	HOMA‐IR	0.61 (0.56–0.66)	2.62	59.8	58.9	0.071	1.45	0.68
	CMI	0.67 (0.62–0.72)	0.76	65.4	62.1		1.73	0.56
Hyperglycemia	HOMA‐IR	0.72 (0.67–0.76)	2.83	70.1	66.8	0.284	2.11	0.45
	CMI	0.69 (0.64–0.74)	0.81	67.3	64.5		1.90	0.51

Abbreviations: AUC, area under the receiver operating characteristic curve; CI, confidence interval; HOMA‐IR, homeostasis model assessment of insulin resistance; CMI, cardiometabolic index. LR + , positive likelihood ratio; LR − , negative likelihood ratio.

* *p* value for the comparison between the AUC of HOMA‐IR and CMI for each outcome (e.g., DeLong test).

ROC analysis showed that the discriminative performance of CMI varied across the individual components of metabolic syndrome and was generally higher than that observed for HOMA‐IR. The highest AUC values for CMI were observed for hypertriglyceridemia (AUC = 0.86), low HDL cholesterol (AUC = 0.84), and central obesity (AUC = 0.79), indicating good discriminatory ability for these metabolic abnormalities. In contrast, the diagnostic performance of CMI was more modest for hypertension and hyperglycemia. Comparison of the ROC curves showed significant differences between CMI and HOMA‐IR for the metabolic components in which CMI displayed the greatest discriminatory capacity, particularly hypertriglyceridemia, low HDL cholesterol, and central obesity. (Table 4).

Reported mean CMI values by number of MetS criteria were 0.52, 0.68, 0.81, 0.93, 1.02 and 1.08 for zero to five criteria, respectively. Because three CMI inputs correspond to MetS components, the pattern is partly inherent in the index and outcome definitions.

## 4 Discussion

This cross‐sectional study found an association between CMI and prevalent MetS in a selected group of postmenopausal women with severe obesity. The reported AUC was numerically higher for CMI than for HOMA‐IR.

These findings reinforce the concept that composite indices integrating anthropometric and biochemical parameters may better reflect the complexity of cardiometabolic risk than isolated markers. Metabolic syndrome is a multifactorial condition characterized by the coexistence of central obesity, insulin resistance, dyslipidemia, and hypertension [1]. CMI shares waist, triglyceride and HDL information with the ATP III outcome definition. This incorporation overlap is a major limitation and may partly explain both its ROC discrimination and its rise across the number of MetS components. The latter pattern does not independently demonstrate severity or prospective risk. Prior studies in women with severe obesity offer context [16], but do not externally validate the present estimate. In this context, Tamini et al. [18] demonstrated that CMI is strongly associated with both the presence and severity of MetS, with ROC areas around 0.82, confirming its high diagnostic accuracy in populations with obesity.

In our study, the strong CMI–waist correlation is expected in part from the waist‐to‐height ratio in the formula. CMI and waist circumference should not be treated as independent measures of visceral fat. It is well established that visceral fat is metabolically active and contributes to insulin resistance, dyslipidemia, and chronic inflammation [6, 7]. Unlike BMI, which does not differentiate between fat and lean mass or account for fat distribution [11, 12]. Correlations with glucose, insulin and HOMA‐IR may reflect concurrent metabolic clustering. Associations with triglycerides and HDL cholesterol are partly mathematical because both analytes are CMI inputs. This is consistent with the study by Zhou et al. [17], who reported a significant positive association between CMI and hyperuricemia, with an odds ratio of 2.62 (95% CI: 1.91–3.61), indicating that higher CMI values are associated with systemic metabolic alterations beyond MetS alone. HOMA‐IR was included as an insulin‐resistance comparator that does not directly contain the triglyceride, HDL and waist criteria. CRP did not differ significantly by MetS status, and its correlation with CMI was weak. Ramuth et al. [24] demonstrated that higher CMI values are associated with increased levels of pro‐inflammatory cytokines such as IL‐6, TNF‐α, and IFN‐γ, particularly in older adults with obesity. This concept of “metaflammation” highlights the interplay between metabolic and inflammatory pathways and may explain the strong association between CMI and adverse clinical outcomes. These prior reports of inflammatory cytokines should not be presented as evidence of an inflammatory mechanism in this cohort.

The cut‐off of 0.84 was selected within the present sample and may be optimistic. A similar published cut‐off does not establish external validity. The referral setting, exclusively Caucasian postmenopausal population and severe obesity criterion limit transportability. Comparison of the ROC curves showed significant differences between CMI and HOMA‐IR for the metabolic components in which CMI displayed the greatest discriminatory capacity, particularly hypertriglyceridemia, low HDL cholesterol, and central obesity. Overall, these findings suggest that the higher diagnostic performance of CMI for metabolic syndrome is mainly driven by its ability to identify abnormalities related to central adiposity and the lipid profile, which is consistent with the components incorporated into the CMI formula

Limitations include the cross‐sectional design, referral‐based selection, lack of external validation, and incorporation overlap between CMI and the MetS definition. The absence of participant‐level data with this revision prevents verification of HOMA‐IR, the paired AUC comparison, confidence intervals for threshold measures, and component‐wise analyses. Medication and menopausal‐hormone‐therapy details, and BIA conditions, require confirmation from source records. Diet and physical activity were not fully assessed. This study has several strengths that enhance the validity of its findings. The relatively large sample size of postmenopausal women with obesity provides adequate statistical power to detect significant associations. The use of standardized protocols for anthropometric and biochemical measurements reduces variability and increases reproducibility. In addition, the comprehensive assessment of multiple metabolic and adiposity parameters allows for a detailed characterization of the study population. The inclusion of ROC curve analysis, likelihood ratios, and Youden index further strengthens the evaluation of the diagnostic performance of CMI.

In conclusion, CMI was associated with prevalent MetS and showed apparent discrimination in this selected sample of postmenopausal women with severe obesity. Its overlap with the diagnostic criteria limits the interpretation of this finding. Independent external validation and prospective studies are required before recommending its routine clinical use.

## Author Contributions

**Daniel Luis:** conceptualization, validation, writing – original draft. **Olatz Izaola:** investigation. **Juan José Lopez Gomez:** investigation, conceptualization. **Daniel Rico:** investigation, methodology. **David Primo:** investigation, validation, formal analysis.

## Funding

The authors have nothing to report.

## Ethics Statement

This study protocol was reviewed and approved by [HCVUA Committee], approval number [No 6/2020]. Written Informed consent was obtained from all individual participants included in the study.

## Consent

Signed informed consent was obtained in all participants. This research complies with the guidelines for human studies in accordance with the World Medical Association Declaration of Helsinki.

## Conflicts of Interest

The authors declare no conflicts of interest.

## Transparency Statement

The lead (Daniel de Luis) affirms that this manuscript is an honest, accurate, and transparent account of the study being reported; that no important aspects of the study have been omitted; and that any discrepancies from the study as planned (and, if relevant, registered) have been explained.

## Data Availability

The data that support the findings of this study are available on request from the corresponding author. The data are not publicly available due to privacy or ethical restrictions.

## References

[1] S. Mottillo , K. B. Filion , J. Genest , et al., “The Metabolic Syndrome and Cardiovascular Risk,” Journal of the American College of Cardiology 56, no. 14 (2010): 1113–1132, 10.1016/j.jacc.2010.05.034. 20863953

[2] A. Misra and L. Khurana , “Obesity and the Metabolic Syndrome in Developing Countries,” Journal of Clinical Endocrinology and Metabolism 93, no. 11 Suppl 1 (2008): 9–30, 10.1210/jc.2008-1595. 18987276

[3] D. M. Thomas , C. Bredlau , A. Bosy‐Westphal , et al., “Relationships Between Body Roundness With Body Fat and Visceral Adipose Tissue Emerging From a New Geometrical Model,” Obesity 21, no. 11 (2013): 2264–2271, 10.1002/oby.20408. PMC3692604 23519954

[4] F. Ebtekar , S. Dalvand , and R. G. Gheshlagh , “The Prevalence of Metabolic Syndrome in Postmenopausal Women: A Systematic Review and Meta‐Analysis in Iran,” Diabetes & Metabolic Syndrome 12, no. 6 (2018): 955–960, 10.1016/j.dsx.2018.06.002. 29891195

[5] S. A. Paschou , K. I. Athanasiadou , and N. Papanas , “Menopausal Hormone Therapy in Women With Type 2 Diabetes Mellitus: An Updated Review,” Diabetes Therapy 15, no. 4 (2024): 741–748, 10.1007/s13300-024-01546-1. PMC10951155 38363540

[6] C. S. Fox , J. M. Massaro , U. Hoffmann , et al., “Abdominal Visceral and Subcutaneous Adipose Tissue Compartments: Association With Metabolic Risk Factors in the Framingham Heart Study,” Circulation 116, no. 1 (2007): 39–48, 10.1161/CIRCULATIONAHA.106.675355. 17576866

[7] C. Walton , B. Lees , D. Crook , M. Worthington , I. F. Godsland , and J. C. Stevenson , “Body Fat Distribution, Rather Than Overall Adiposity, Influences Serum Lipids and Lipoproteins in Healthy Men Independently of Age,” American Journal of Medicine 99, no. 5 (1995): 459–464, 10.1016/S0002-9343(99)80220-4. 7485201

[8] National Institutes of Health . Clinical Guidelines on the Identification, Evaluation, and Treatment of Overweight and Obesity in Adults: The Evidence Report,” Obesity Research 6 Suppl 2 (1998): 51S–209S. 9813653

[9] T. Pischon , H. Boeing , K. Hoffmann , et al., “General and Abdominal Adiposity and Risk of Death in Europe,” New England Journal of Medicine 359, no. 20 (2008): 2105–2120, 10.1056/NEJMoa0801891. 19005195

[10] C. Prospective Studies Collaboration , “Body‐Mass Index and Cause‐Specific Mortality in 900 000 Adults: Collaborative Analyses of 57 Prospective Studies,” Lancet 373, no. 9669 (2009): 1083–1096, 10.1016/S0140-6736(09)60318-4. PMC2662372 19299006

[11] J. Gómez‐Ambrosi , C. Silva , J. C. Galofré , et al., “Body Mass Index Classification Misses Subjects With Increased Cardiometabolic Risk Factors Related to Elevated Adiposity,” International Journal of Obesity 36, no. 2 (2012): 286–294, 10.1038/ijo.2011.100. 21587201

[12] J. Bigaard , K. Frederiksen , A. Tjønneland , et al., “Body Fat and Fat‐Free Mass and All‐Cause Mortality,” Obesity Research 12, no. 7 (2004): 1042–1049, 10.1038/oby.2004.131. 15292467

[13] S. M. Kang , J. W. Yoon , H. Y. Ahn , et al., “Android Fat Depot Is More Closely Associated With Metabolic Syndrome Than Abdominal Visceral Fat in Elderly People,” PLoS One 6, no. 11 (2011): e27694, 10.1371/journal.pone.0027694. PMC3214067 22096613

[14] J. C. Seidell , “Waist Circumference and Waist‐Hip Ratio in Relation to All‐Cause Mortality, Cancer and Sleep Apnea,” European Journal of Clinical Nutrition 64, no. 1 (2010): 35–41, 10.1038/ejcn.2009.71. 19639001

[15] H. Petursson , J. A. Sigurdsson , C. Bengtsson , T. I. L. Nilsen , and L. Getz , “Body Configuration as a Predictor of Mortality: Comparison of Five Anthropometric Measures in a 12‐year Follow‐Up of the Norwegian HUNT 2 Study,” PLoS One 6, no. 10 (2011): e26621, 10.1371/journal.pone.0026621. PMC3197688 22028926

[16] S. Lazzer , M. D'Alleva , M. Isola , et al., “Cardiometabolic Index (CMI) and Visceral Adiposity Index (VAI) Highlight a Higher Risk of Metabolic Syndrome in Women With Severe Obesity,” Journal of Clinical Medicine 12, no. 9 (2023): 3055, 10.3390/jcm12093055. PMC10179486 37176497

[17] Y. Zhou , Y. Zou , Y. Cao , et al., “The Association Between the Cardiometabolic Index and Hyperuricemia: 2011–2016 NHANES,” Frontiers in Endocrinology 16 (2025): 1545968, 10.3389/fendo.2025.1545968. PMC12106002 40433413

[18] S. Tamini , A. Bondesan , D. Caroli , and A. Sartorio , “The Lipid Accumulation Product Index (LAP) and the Cardiometabolic Index (CMI) Are Useful for Predicting the Presence and Severity of Metabolic Syndrome in Adult Patients With Obesity,” Journal of Clinical Medicine 13, no. 10 (2024): 2843, 10.3390/jcm13102843. PMC11122168 38792386

[19] C. Liu , Z. Zhang , T. Meng , et al., “Correlation of Cardiometabolic Index (CMI) With Postmenopausal Depression: A Cross‐Sectional Study Based on NHANES 2005–2018,” Women & Health 65, no. 8 (2025): 706–718, 10.1080/03630242.2025.2570176. 41047515

[20] Expert Panel on Detection, Evaluation and Treatment of High Blood Cholesterol in Adults (Adult Treatment Panel III) ., “Executive Summary of the Third Report of the National Cholesterol Education Program (NCEP),” Journal of the American Medical Association 285 (2001): 2486–2497. 10.1001/jama.285.19.2486 11368702

[21] H. Lukaski , P. Johnson , W. Bolonchuk , and G. Lykken , “Assessment of Fat‐Free Mass Using Bioelectrical Impedance Measurements of the Human Body,” American Journal of Clinical Nutrition 41 (1985): 810–817, 10.1093/ajcn/41.4.810. 3984933

[22] W. T. Friedewald , R. I. Levy , and D. S. Fredrickson , “Estimation of the Concentration of Low‐Density Lipoprotein Cholesterol in Plasma Without Use of the Preparative Ultracentrifuge,” Clinical Chemistry 18 (1972): 499–502, 10.1093/clinchem/18.6.499. 4337382

[23] J. P. Hosker , D. R. Matthews , A. S. Rudenski , et al., “Continuous Infusion of Glucose With Model Assessment: Measurement of Insulin Resistance And?‐Cell Function in Man,” Diabetologia 28 (1985): 401–411, 10.1007/BF00280882. 3899824

[24] H. Ramuth , K. Narayanasamy , R. Murugesan , S. Periyasamy , P. Rathinavelu , and S. Subramanian , “Association of Cardiometabolic Index With Inflammatory Markers in Older Adults With Obesity,” Journal of Inflammation Research 16 (2023): 1235–1245.

